# The performance of Xpert MTB/RIF and MTBDRplus within a Programmatic setting at TB Laboratory in Rio de Janeiro, Brazil

**DOI:** 10.1590/0037-8682-0167-2024

**Published:** 2024-09-13

**Authors:** Thiago da Silva Santos Malaquias, Eunice Petris Ribeiro, Tatiana Cristina Pereira Dutra, Marina Ricardo, Richard Salvato, Marcela Bhering, Daniella Castanheira Bartholomeu, Elis Regina Dalla-Costa, Miguel Viveiros, Elisangela Costa da Silva, Afrânio Kritski

**Affiliations:** 1 Universidade Federal do Rio de Janeiro, Faculdade de Medicina, Programa Acadêmico de Tuberculose, Rio de Janeiro, RJ, Brasil.; 2 Secretaria Estadual da Saúde do Rio Grande do Sul, Centro de Desenvolvimento Científico e Tecnológico, Porto Alegre, RS, Brasil.; 3 Universidade Federal do Rio Grande do Sul, Centro de Biotecnologia, Programa de Pós-Graduação em Biologia Celular e Molecular, Porto Alegre, RS, Brasil.; 4 Fundação Oswaldo Cruz, Escola Nacional de Saúde Pública Sergio Arouca, Rio de Janeiro, RJ, Brasil.; 5 Universidade Federal de Minas Gerais, Instituto de Ciências Biológica, Departamento de Parasitologia, Belo Horizonte, MG, Brasil.; 6 Universidade Nova de Lisboa, Instituto de Higiene e Medicina Tropical, Lisboa, Portugal.; 7 Universidade Estadual do Norte Fluminense Darcy Ribeiro, Centro de Biociência e Biotecnologia, Laboratório de Biologia do Reconhecer, Campos dos Goytacazes, RJ, Brasil.; 8 Secretaria de Estado de Saúde do Rio de Janeiro, Fundação Saúde, Rio de Janeiro, RJ, Brasil.

**Keywords:** Drug-Resistant Tuberculosis, Molecular Diagnostic Techniques, Sensitivity and Specificity, Diagnosis, Mycobacterium tuberculosis

## Abstract

**Background:**

Few studies in routine settings have confirmed the high accuracy of the Xpert MTB/RIF assay for detecting rifampicin resistance (RR) and the first-line probe assay (FL-LPA) for detecting both RR and isoniazid resistance (INH^R^).

**Methods:**

The performance of Xpert MTB/RIF and MTBDRplus VER 2.0 LPA was evaluated in 180 *Mycobacterium tuberculosis* samples collected from January 2018 to December 2019 in Rio de Janeiro, Brazil. The results were compared with those from BACTEC MGIT 960 culture and drug susceptibility testing (DST). Whole-genome sequencing was performed on the samples with discordant results.

**Results:**

The Xpert MTB/RIF assay showed a sensitivity (Se) of 93.3% and a specificity (Sp) of 97.6%, detecting RR. The performance of FL-LPA to identify RIF and INH resistance was, respectively, (Se) 100% and 83.3% and (Sp) 98.8% and 100%. Among 18 clinical isolates with INH^R^ detected by FL-LPA, mutations in the *katG* gene were observed in 100% of samples, of which only two (11.1%) had mutations in both *katG* and *inhA* genes. Overall, the discordant results were identified in 9 (5%) samples. Among the four Xpert RIF-resistant and DST-sensitive, two harbored mutations in rpoB Leu430Pro. Among the four FL-LPA-sensitive and DST-resistant, one had a mutation in *inhA* 17G>T. FL-LPA showed high accuracy in detecting RR and INH^R^.

**Conclusions:**

The MTBDRplus test demonstrated excellent performance in detecting RR, and INH^R^ in clinical isolates under routine conditions at a reference laboratory in Rio de Janeiro, Brazil. Incorporating both tests can improve drug-resistant tuberculosis treatment outcomes and monitor the INH^R^ incidence.

## INTRODUCTION

The 2022 World Health Organization (WHO) Global Report states that in 2021, tuberculosis (TB) will become the second leading cause of death due to infectious diseases, following COVID-19, surpassing HIV/AIDS^1^. While drug-sensitive tuberculosis (DS-TB) is effectively cured within 6 to 9 months with the WHO recommended 1^st^ line multiple anti-TB drug regimen, for TB patients with *Mycobacterium tuberculosis* (MTB) resistant to both isoniazid and rifampicin, defined as multidrug-resistant tuberculosis (MDR-TB), treatment can take up to two years using second-line drugs that are frequently more expensive, toxic, and have low favorable outcome^1^. The burden of drug-resistant tuberculosis (DR-TB) also increased by 3% between 2020 and 2021, with 450,000 new cases of rifampin-resistant (RR) in 2021^1^. 

In addition, WHO provided global estimates of the incidence of isoniazid monoresistance (INH^R^) for the first time: there were 1.4 million incident cases of INH^R^-TB, of which 1.1 million were susceptible to rifampicin^1^. Most of these patients were not diagnosed with DR TB and did not receive appropriate treatment. Furthermore, there are limited data available on TB treatment outcomes among patients with INH^R^-TB in high-burden countries^2^. 

MTB culture-based methods (solid or liquid), the gold standard for TB diagnosis, usually take several weeks and require empirical treatment without TB drug resistance results. To improve the early detection of MDR/RR-TB and reduce the time for initiation of appropriate treatment, the WHO recommended the following molecular tests: Genotype MTBDRplus/First Line - Line Probe Assay (henceforth FL-LPA) (Hain Lifescience, Nehran, Germany) in 2008 and Xpert MTB/RIF (Cepheid, USA) in 2010^3^.

The meta-analysis of the Xpert MTB/RIF used for DR-TB diagnosis confirmed that it can be reliably executed directly on a respiratory sample in less than one day, with a sensitivity of 67 to 89% and high specificity, above 95%, and it also allows the direct detection of RR with high accuracy^4^. 

As the detection of INH^R^ by molecular tests is not usually possible in low- and middle-income countries, the identification of RR by Xpert MTB/RIF has been used as a predictive marker of MDR-TB, assuming that, in a certain region, there is a low prevalence of INH^R^ ^5^. 

Therefore, FL-LPA is recommended for identifying RR and INH^R^, particularly in regions with a high prevalence of mono INH^R^. The high accuracy of FL-LPA has been reported by meta-analysis; for RR, it showed a pooled sensitivity and specificity (with 95% confidence intervals) of 96.7% (95.6-97.5%) and 98.8% (98.2-99.2%), respectively; and for INH^R^, it demonstrated sensitivity and specificity of 90.2% (88.2-91.9%) and 99.2% (98.7-99.5%), respectively^6^. However, performance studies in the programmatic settings of Xpert MTB/RIF and FL-LPA for detecting RR, INH^R^, or MDR-TB are limited, especially in high-TB burden and low-middle-income countries^7^ ^-^ ^13^. Thus, the main objective of the present study was to evaluate the performance of Xpert MTB/RIF and FL-LPA for the direct detection of RR, INH^R^, and MDR-TB under routine diagnostic conditions in a laboratory environment in Rio de Janeiro, Brazil, a high-burden DR-TB setting.

## METHODS

### ● Study Design and Population

This was a retrospective data analysis of the performance of Xpert MTB/RIF (Cepheid, USA) and Genotype MTBDRplus VER 2.0/FL-LPA (Hain Lifesciences, Nehren, Germany) on 180 MTB isolates obtained between 2018 and 2019. The analysis was conducted at the Molecular Mycobacteriology Laboratory (LMM) of the Clementino Fraga Filho University Hospital (HUCFF) and the Thorax Diseases Institute (IDT) located in the State of Rio de Janeiro, which has the second highest incidence, highest TB mortality rate, and highest number of DR-TB cases in the country^14^. 

All MTB isolates were obtained from patients with presumed pulmonary TB evaluated at primary health centers in Rio de Janeiro or Duque de Caxias.

### ● MTB isolation and drug-sensitivity testing (DST)

In this study, MTB isolates obtained from respiratory samples were inoculated into an automated liquid culture. Decontamination, inoculation, and incubation of the samples were performed using a BACTEC MGIT 960 system (Becton Dickinson, USA) according to the manufacturer's instructions. 

Samples were decontaminated using the N-acetyl-L-cysteine/sodium hydroxide (NALC-NaOH, 2% NaOH) method. Part of the sediment was resuspended in 3 ml of phosphate buffer (pH 6.8) and directly inoculated into the liquid culture medium. Another part was enriched by culturing in Löwenstein-Jensen culture medium, cryopreserved using a freezing solution (7H9 + 10% glycerol and 10% OADC in liquid medium), and afterwards, defrosted and inoculated into liquid medium. All cultures were maintained at 37°C in the BACTEC MGIT 960 system. 

First-line phenotypic drug sensitivity tests (DST) were also performed using the automated BACTEC MGIT 960 system according to the manufacturer's instructions. Drugs evaluated were streptomycin- SM (1.0 μg/ml), isoniazid- INH (0.1 μg/ml), rifampicin- RIF (1.0 μg/ml), ethambutol- EMB (5.0 μg/ml). DST results for RIF and INH (resistant or susceptible to the critical concentration tested) were considered the gold standard for the evaluation of genotypic results obtained using the Xpert MTB/RIF (Cepheid, USA) and Genotype MTBDRplus/FL-LPA (Hain Lifescience, Nehran, Germany) assays.

### ● Xpert MTB RIF test

Xpert MTB/RIF was performed with a volume of 1.5 ml taken from the clinical sample. The 1.5 ml volume was placed in a 15 ml conical tube, and the sample reagent was added at a ratio of two reagent volumes to one sample volume (2:1) and shaken vigorously 10-20 times, with or without vortex. The tube was incubated for 10 min at room temperature, vortexed vigorously 10-20 times, and incubated for an additional 5 min at room temperature. Samples that were not fully liquefied were shaken again and left at room temperature for 5-10 minutes. Decontamination and liquefaction steps did not exceed 35 min. For the liquefied samples, 2 ml of the total volume was transferred from inside the conical tube and deposited inside the Xpert MTB/RIF cartridge, and the test was performed automatically in a GeneXpert machine^3^.

### ● GenoType®MTBDRplus Kit PCR reaction.

All samples that presented a valid Xpert MTB/RIF test detection were subsequently evaluated using GenoType® MTBDRplus VER 2.0 Kit (FL-LPA). FL-LPA was performed on all sampled MTB isolates, one per patient, using the MGIT DST results. Cultures were subjected to DNA extraction one day before entering the MGIT 960 system instrument for DST. DNA was extracted from the liquid cultures using a Genolyse kit (version 1.0; Hain). The reactions detected on the strips were visually interpreted using a cardboard template. In the case of invalid results, such as no signal with a conjugate or any of the other control probes, and doubtful reactions as weak signals with the gene bands, the test was repeated using a new DNA extraction^6^.

### ● Whole genome sequencing (WGS)

WGS was performed only for samples that showed discordant results between the genotypic and phenotypic assays. DNA from the four samples was subjected to Next-Generation Sequencing (NGS) to obtain the whole-genome sequence. Paired-end sequencing (2 × 150 bp) was performed on an Illumina NextSeq machine using either a 300 cycle v2 mid-output or high-output kit (Illumina, Code FC-404-2003 or Code FC- 404-2004) under standard Illumina® procedure as previously described^15^. 

### ● Statistical analysis

Kappa concordance index (K) statistics were calculated based on (a) the proportion of RIF and/or INH results reported as resistant versus (b) the proportion of RIF and INH results reported as susceptible, using each of the genotypic molecular methods, Xpert MTB/RIF and FL-LPA, compared to the respective DST results obtained by the gold standard, the BACTEC MGIT 960 phenotypic method. The sensitivity (Se), specificity (Sp), positive predictive value (PPV), negative predictive value (NPV), and accuracy (A) of the Xpert MTB/RIF and FL-LPA tests were calculated from the ratio of genotypic results to phenotypic gold-standard results. 

WGS bioinformatics analysis of the raw reads was conducted as previously described by Salvato et al^15^. SPSS software, version 21.0, was used for the kappa index analyses. The remaining statistical analyses were performed using the online software MedCalc Software - Diagnostic Test Evaluation Calculator (version 20.027), available at https://www.medcalc.org/calc/diagnostic_test.php.

## RESULTS

### ● Resistance detected by First Line - Line Probe Assay

The resistance profiles of 180 clinical samples were analyzed using Xpert MTB/RIF, FL-LPA, and drug susceptibility testing after isolation in a liquid medium (MGIT 960). 

The performance of Xpert MTB/RIF in detecting RR showed a sensitivity (Se) of 93.3% and a specificity (Sp) of 97.6% (Table 1). The performance of FL-LPA in identifying RR and INH^R^ was 100% and 83.3% for sensitivity and 98.8% and 100% for specificity, respectively. The performance of FL-LPA in detecting MDR-TB was high, with a PPV of 93.3%.

**TABLE 1:** Performance of Xpert MTB RIF and MTBDRplus assays for tuberculosis and for rifampicin and isoniazid resistance detection at reference laboratory (n=180).

	True positive	False Positive	False Negative	True Negative	Sensitivity	Specificity	Positive Predicitve Value	Negative Predictive Value	Kappa value
					(95% CI)	(95% CI)	(95% CI)	(95% CI)	(95% CI)
**RIF resistance**									
Xpert MTB RIF	14	4	1	161	93.3%	97.6%	77.8%	99.4%	0.833
					(68.1-99.8)	(93.9-99.3)	(56.8-90.3)	(96.0-99.9)	(0.759-0.908)
MTBDRplus	15	2	0	163	100%	98.8%	88.24%	100%	0.931
					(78.2-100)	(95.7-99.9)	(65.4-96.8)	(97.8-100)	(0.857-1.000)
**INH resistance**									
MTBDRplus	20	0	4	156	83.3%	100%	100%	97.5%	0.887
					(62.6-95.3)	(97.7- 100)	(83.2-100)	(94.1-98.7)	(0.818-0.956)
**MDR-TB resistance**									
MDTDRplus	14	1	0	165	100%	99.4%	93.3%	100%	0.963
					(76.8-100)	(96.7-99.9)	(66.5-99.9)	(97.7-100)	(0.889-1000)

**RIF:** rifampicin; **INH:** isoniazide; **MDR:** multidrug-resistant; **CI:** Confidence interval.

Compared to MGIT 960 phenotypic DST, a high agreement with FL-LPA and Xpert MTB/RIF for RR was found - 0.93 and 0.83, respectively, and for INH^R^ with FL-LPA, 0.88. The agreement between Xpert MTB/RIF and FL-LPA for RR detection was 0.91. Monoresistance to SM, INH, and RIF was found respectively in 10%, 10%, and 0.6% of the samples. Ethambutol resistance was detected in one MDR-TB case. 

Among the 11 samples with RR, the most prevalent mutations in the *rpoB* gene were in Wt 8 (codons 530-533) and Wt 7 (codons 526-529), representing 60% and 10%, respectively. 

Among the 18 samples with INH^R^ detected by FL-LPA, a mutation in the *katG* gene was observed in 100% of the samples, with only two (10%) having mutations in both *katG* and *inhA* genes. None of the samples harbored mutations in the *inhA* gene alone. In addition, only one INH^R^ sample showed both WT1 (wild type) and mutant bands, suggesting heteroresistance or mixed infection.

### ● Discordant results analysis

Overall, discordance between genotypic and phenotypic results was identified in nine (5.0%) samples (Table 2): discordance between Xpert MTB/RIF and MGIT-960 DST in five cases, four cases which Xpert MTB/RIF indicated resistance and MGIT 960 DST showed susceptible and one case where Xpert MTB/RIF indicated rifampicin susceptibility, but the MGIT 960 DST showed resistance. For the four Xpert MTB/RIF resistant discordant cases, two had a Leu430Pro at *rpoB*, which is a borderline rifampicin resistance mutation that accounts for highly discordant results in phenotypic DST^16^. FL-LPA for RIF resistance was discordant with MGIT 960 DST in two cases, and no WGS results were available. Four samples with FL-LPA susceptible to INH and MGIT 960 DST resistant to INH showed one mutation in hA 17G>T, which is an INH^R^ canonical mutation in the inhA promoter region^17^.

**TABLE 2:** Evaluation of discordant results by whole genome sequencing and clinical history.

Nº MTB Isolate	RIF (Xpert)	RIF MTBDR plus	RIF (MGI T)	INH MTBDR plus	INH (MGIT)	WGS (rpoB)	WGS (katG/ inhA)
2640/18	S	R	R	R	R	Ser450Leu	-
747/18	R	S	S	R	R	Leu430Pro	-
17/19	R	S	S	R	R	Leu430Pro	-
2112/19	R	R	S	R	R	-	-
485/18	R	R	S	S	S	-	-
1191/18	S	S	S	S	R	-	-
2427/18	S	S	S	S	R	-	-
475/18	S	S	S	S	R	-	-
451/18	S	S	S	S	R	-	inhA-17G>T

**N:** Number; **RIF:** Rifampicin; **INH:** Isoniazid; **WGS:** whole genome sequencing; **S:** Susceptible; **R:** Resistant.

Comparing Xpert MTB RIF and FL-LPA results, among the three samples with discordant RIF resistance results, MGIT SIRE confirmed the FL-LPA results in one RIF-resistant sample and two RIF-sensitive samples.

## DISCUSSION

The Xpert MTB/RIF test for clinical samples and the FL-LPA molecular test for MTB clinical isolates showed high accuracy in the early diagnosis of RR/INH^R^ and MDR-TB when used under routine diagnostic conditions in Rio de Janeiro, Brazil. Although the predictive values may vary according to the prevalence of the disease in different settings^18^, the sensitivity and specificity of the FL-LPA molecular assay obtained in this study are in accordance with the literature for high-burden settings in low- and middle-income countries under field conditions^7^ ^-^ ^13^ ^,^ ^19^. The performance of Xpert MTB/RIF to detect RR was also high, showing a sensitivity (Se) of 93.3% and a specificity (Sp) of 97.6%, similar to those described elsewhere^3^. In addition, compared to MGIT 960 phenotypic DST, a high agreement with FL-LPA and Xpert MTB/RIF for RR was found (0.93 and 0.83, respectively), and 0.88 for INH^R^ with FL-LPA. 

FL-LPA was superior to Xpert MTB/RIF in the detection of RR, which is consistent with previously published data^20^. This is likely because FL-LPA detection is performed on DNA isolated from cultures, which yields high quantities of the template and high sensitivity (100% and 92.9%, respectively)^21^. 

Furthermore, FL-LPA can detect RR and INH^R^ directly in clinical samples, irrespective of the smear status, without the need for MTB culture growth, as highlighted in other series^7^ ^,^ ^8^ ^,^ ^10^ ^,^ ^12^ ^,^ ^22^. It is important to accelerate decision-making by the clinical team in defining the appropriate treatment course for RIF resistance, INH resistance, and MDR-TB. Thus, FL-LPA with Xpert MTB/RIF may be helpful in regions with a high prevalence of INH resistance that is not detected by Xpert^3^. The discordant results among genotypic and phenotypic tests were identified in only nine (5.0%) samples. In addition, the discordant results between Xpert MTB/RIF and FL-LPA for RIF resistance were identified in only three samples, confirmed by MGIT SIRE the FL-LPA results in 1 RIF resistant and 2 RIF sensitive samples.

In our study, false rifampicin resistance was detected by Xpert in four samples and by FL-LPA in two samples. These results may be associated with mutations in the *rpoB* gene, including what others have referred to as 'disputed mutations' or silent mutations^23^ ^-^ ^25^. Notably, among the four Xpert RR and DST sensitive cases, two showed mutations in rpoB Leu430Pro similar to those described by Brandao et al. in São Paulo, Brazil^24^, and different from those described by Abanda et al., in Cameroon^23^ and by Miotto et al^25^ in Italy. Being part of the “RIF^R^ disputed” mutations (L430P, D435Y, H445C/L/N/S, and L452P), MTB isolates carrying this genetic profile are known to have a slow growth delta, taking about 30 days, in liquid culture medium with the drug rifampicin. Therefore, resistance was not detected in phenotypic testing, which was completed within 12 days according to the MGIT 960 base protocol^23^ ^-^ ^25^. 

Using Xpert MTB RIF results, as recommended by WHO, as an indicator of MDR^3^, a low rate of discrepancy between genotypic and phenotypic results is expected, as the clinical staff could start RR/MDR-TB treatment. However, in São Paulo State, a high false RR resistant results rate (55%) was described by Brandao et al^24^ and was associated with unusual clusters of rpoB mutations largely associated with low resistance levels. Unfortunately, in countries with a high TB burden, data on the clinical and economic impact of Xpert and/or MTBDRplus under field conditions^26^ ^-^ ^28^ is scarce. In a nationwide study, Villalva-Serra K et al^27^ described that the Xpert implementation in Brazil resulted in a 9.7% increase in TB notification and substantial improvements in DR-TB (63.6%) detection compared to expected notifications if it had not been implemented^26^ showed in a pragmatic clinical trial, compared to the MGIT group, physicians received the genotypic DST result earlier using Xpert MTB RIF than those using MGIT (median 7.0 vs. 55.5 days; p<0.01), and using MTBDR plus in MTB isolate (30.0 vs. 40.2 days, p<0.01). Culture conversion after six months was higher for Xpert (90.9% vs. 79.3%, p=0.39) and not for LPA (80.0% vs. 83.0%, p=0.81). Soares et al^28^, comparing the activity-based costs (ABC) using phenotypic and genotypic DST in a reference laboratory showed that ABC were higher for MGIT SIRE (US$ 136.80) and lower for Genotype® MTBDRplus (US$ 48.38) and for Xpert® MTB/RIF (US$ 9.89). Therefore, interactions between public managers, clinicians, and laboratory technicians are urgently needed to provide a more rapid and precise diagnostic algorithm at the local level and appropriate TB treatment initiation.

In the analysis of discordant results for INH^R^, four samples showed sensitivity in FL-LPA and resistance in the phenotypic test; among these samples, one was confirmed to have the mutation inhA-17G>T. Such false-negative results can be explained by the analysis profile of the FL-LPA test, which features only two detection genes for INH^R^. Similar results have been described previously and attributed to the amplification of DNA released from non-viable bacilli in cases of heteroresistance^10^. Furthermore, it is well known that about 10% to 25% of INH^R^ strains are thought to have mutations outside *katG* and *inhA* loci, which, regrettably, we were not able to detect^29^. The molecular mechanisms underlying INH^R^ involve several genes in multiple networks and biosynthetic pathways. The recent association between efflux pumps, other genes, and INH^R^ has also gained considerable attention^30^. Substitutions were observed in fabG1, fabD, nat, accD6, and fbpC as reported by Unissa, et al^31^. Understanding the mechanisms associated with INH^R^ would allow better detection of INH^R^. This information will aid in the design of new drug strategies. 

Overall, *rpo*B Ser531Leu and *kat*G Ser315Thr mutations were predominant in our study, whereas inhA mutations were found in a small number of cases, similar to other settings^19^ ^,^ ^22^ ^,^ ^24^ ^,^ ^32^ ^-^ ^34^. These genetic markers show high accuracy, as we found in our study, where 93.3% of the RR samples detected by Xpert were characterized as MDR-TB by phenotypic DST. We also observed a low rate (0.5%) of probable heteroresistance (the clinical relevance of which is unknown), as described by Kumar et al^35^ and Figueiredo et al^36^. Among the weaknesses of this study, we cite the limitation of WGS coverage on all discordant samples complemented by clinical data and the absence of MIC determination, as it is not part of the routine procedure, which determines the levels of resistance, especially for borderline mutations.

In conclusion, the MTBDRplus showed excellent performance as a rapid molecular test for the detection of RR-, INH^R^, and MDR-resistant TB in clinical isolates under routine use in a reference laboratory in Rio de Janeiro, Brazil. Considering the low proportion of discordant results in the detection of RIF resistance between Xpert MTB RIF and MTBDRplus, such tests should be incorporated into the routine diagnosis of drug-resistant TB in regions with high DR-TB burden, as they expedite and support staff in choosing the appropriate clinical therapeutic approach for patients with TB, thus promoting a lower proportion of unfavorable TB treatment outcomes. Of special importance is the ability to routinely detect mono INH^R^ by FL-LPA and the need to follow up on these cases as their outcomes and progression towards MDR-TB are barely known, and their incidence is increasing dramatically in Brazil and worldwide^37^.

## Data Availability

*Mycobacterium tuberculosis* WGS data are available in the NCBI BioProject ID PRJNA719107.
